# Targeting neddylation induces DNA damage and checkpoint activation and sensitizes chronic lymphocytic leukemia B cells to alkylating agents

**DOI:** 10.1038/cddis.2015.161

**Published:** 2015-07-09

**Authors:** C Paiva, J C Godbersen, A Berger, J R Brown, A V Danilov

**Affiliations:** 1 Knight Cancer Institute, Oregon Health and Science University, Portland, OR, USA; 2 Geisel School of Medicine at Dartmouth, Hanover, NH, USA; 3 Millennium Pharmaceuticals, Inc., a wholly owned subsidiary of Takeda Pharmaceutical Company Ltd, Cambridge, MA, USA; 4 Medical Oncology, Dana-Farber Cancer Institute, Boston, MA, USA

## Abstract

Microenvironment-mediated upregulation of the B-cell receptor (BCR) and nuclear factor-*κ*B (NF-*κ*B) signaling in CLL cells resident in the lymph node and bone marrow promotes apoptosis evasion and clonal expansion. We recently reported that MLN4924 (pevonedistat), an investigational agent that inhibits the NEDD8-activating enzyme (NAE), abrogates stromal-mediated NF-*κ*B pathway activity and CLL cell survival. However, the NAE pathway also assists degradation of multiple other substrates. MLN4924 has been shown to induce DNA damage and cell cycle arrest, but the importance of this mechanism in primary neoplastic B cells has not been studied. Here we mimicked the lymph node microenvironment using CD40 ligand (CD40L)-expressing stroma and interleukin-21 (IL-21) to find that inducing proliferation of the primary CLL cells conferred enhanced sensitivity to NAE inhibition. Treatment of the CD40-stimulated CLL cells with MLN4924 resulted in deregulation of Cdt1, a DNA replication licensing factor, and cell cycle inhibitors p21 and p27. This led to DNA damage, checkpoint activation and G2 arrest. Alkylating agents bendamustine and chlorambucil enhanced MLN4924-mediated DNA damage and apoptosis. These events were more prominent in cells stimulated with IL-21 compared with CD40L alone, indicating that, following NAE inhibition, the culture conditions were able to direct CLL cell fate from an NF-*κ*B inhibition to a Cdt1 induction program. Our data provide insight into the biological consequences of targeting NAE in CLL and serves as further rationale for studying the clinical activity of MLN4924 in CLL, particularly in combination with alkylating agents.

The ubiquitin–proteasome system ensures timely destruction of intracellular proteins. In the past decade, protein degradation has become a pharmacologic target: proteasome inhibitors (e.g., bortezomib) are currently being used in therapy of plasma and B-cell neoplasms. Inhibiting the ubiquitination process upstream of the proteasome represents a promising alternative approach. In this regard, ubiquitin-like modifiers (Ubl) such as NEDD8, ISG15 (interferon-stimulated gene 15), and SUMO (small ubiquitin-like modifier) regulate diverse cellular processes, depending on the exact Ubl and substrate involved. One such Ubl, NEDD8, modulates Cullin-RING E3 ubiquitin ligase (CRL) activity through covalent modification, neddylation.^1^

Chronic lymphocytic leukemia (CLL) B cells are highly dependent on cell–cell interactions in the lymph node and bone marrow microenvironment.^2^ Stromal-mediated upregulation of B-cell receptor (BCR) and nuclear factor-*κ*B (NF-*κ*B) signaling in CLL cells resident in these niches ensures apoptosis evasion and promotes proliferation and clonal expansion.^3^ We recently reported that MLN4924 (pevonedistat), an investigational inhibitor of the NEDD8-activating enzyme (NAE), successfully abrogates NF-*κ*B pathway activity, CLL cell survival and chemoresistance in an *in vitro* co-culture model that mimics the lymph node microenvironment.^4^ NAE adenylates NEDD8 at its C-terminus and allows its transfer to a specific cysteine within NAE, thus initiating a process of neddylation. Active NEDD8 is then transferred to the cysteine of the ubiquitin-conjugating enzyme (E2) specific for the pathway (Ubc12), and is finally conjugated to the CRLs.^5^ CRLs are responsible for ubiquitination and degradation of their substrate proteins. NAE–NEDD8 interaction is disrupted when a covalent adduct is formed between NEDD8 and MLN4924.^6^ Ultimately, this prevents ubiqitination of CRL target proteins, extending their half-life, thereby increasing levels of inhibitor of NF-*κ*B (I*κ*B), a negative pathway modulator.^6, 7, 8^ However, CRLs process a variety of proteins that, in addition to signal transduction (I*κ*B*α*, DEPTOR, *β*-catenin, hypoxia-inducible factor-1*α*) and apoptosis (NOXA, Bim_EL_), are important regulators of cell cycle and DNA replication (e.g., p21^Cip1^, p27^Kip1^, Wee1, Cyclin D1 and Cdt1).^9, 10, 11, 12, 13, 14^ Because of the diversity of CRL target substrates, the biological consequences of their inhibition are tissue dependent. In adherent solid tumor cell lines, inhibition of neddylation resulted in characteristic deregulation of cell cycle with DNA re-replication, checkpoint activation and cell cycle arrest, thought to be secondary to stabilization of the replication-licensing protein Cdt1 (chromatin licensing and DNA replication factor 1) and cyclin-dependent kinase (CDK) inhibitor p21^Cip1^.^11, 15, 16, 17^ However, the importance of this mechanism in primary neoplastic B cells has not been studied. Here we determined that, under the conditions promoting cell replication and growth, MLN4924 induces checkpoint activation and cell cycle arrest in primary CLL B -cells. This mechanism complements abrogation of NF-*κ*B pathway activity to induce apoptosis in CLL.

## Results

### CLL cells induced to proliferate exhibit greater sensitivity to MLN4924

**Figure 1:**
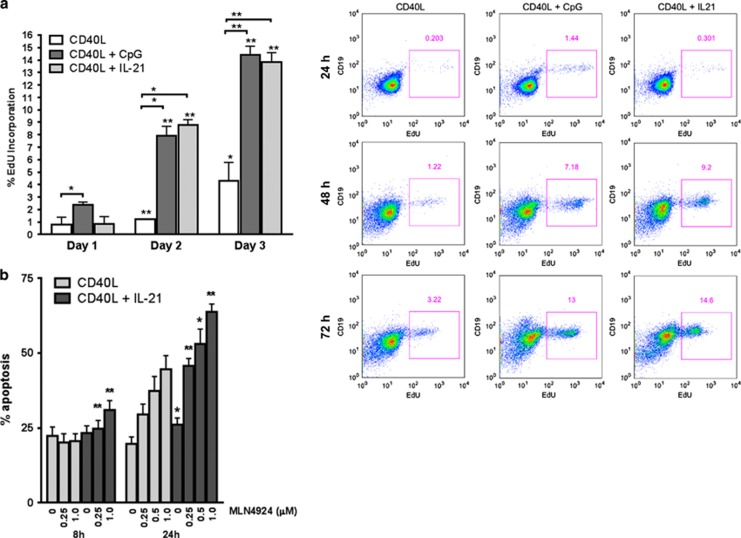
CLL cells induced to proliferate are sensitized to MLN4924. (**a**) CLL cells (*N*=4) were co-cultured with CD40L-expressing stroma for up to 72 h in the presence or not of 25 ng/ml IL-21 or 1.5 *μ*M CpG-ODN. Cells were collected daily, labeled with 5-ethynyl-2′-deoxyuridine (EdU) and assayed by flow cytometry. Data are mean±S.E. A representative image is shown. (**b**) CLL cells were co-cultured with CD40L-expressing stroma in the presence or not of 25 ng/ml IL-21. After 72 h, cells were treated with 0.25–1 *μ*M MLN4924 or vehicle control for the indicated time intervals. Apoptosis within the CD19^+^ subset of cells (*N*=10) was determined by Annexin V and 7-AAD staining. *P<0.05 and **P<0.01 compared with CD40L only

Growth restriction and apoptosis following inhibition of NEDD8-activating enzyme in solid tumor cell lines occurs because of DNA re-replication and damage, yet it is not known whether this occurs in primary lymphoid tumors. CLL cells obtained from peripheral blood rest in G_0_/G_1_ and undergo spontaneous apoptosis when devoid of their supportive microenvironment. Hence, to mimic the lymph node microenvironment and induce CLL cell proliferation *in vitro*, we deployed co-cultures with CD40 ligand (CD40L)-expressing stroma, as previously described by us and others.^4, 18^ Under these conditions, <1% of cells entered S phase as early as 24 h, as evidenced by EdU incorporation (Figure 1a). We then further induced CLL cell proliferation by means of CpG oligodeoxynucleotide (CpG-ODN), a Toll-like receptor agonist,^19^ or interleukin-21 (IL-21). Whereas IL-21 induced apoptosis of the CLL cells outside of their protective niche,^20^ CD40L-stimulated CLL cells may be induced to proliferate in its presence.^21^ Despite intersample variation at earlier time points, up to 20% of CLL cells proliferated after a continuous 72 h exposure to either 1.5 *μ*M CpG-ODN or 25 ng/ml IL-21 (Figure 1a).

To determine whether NAE inhibition influences survival of the CLL cells that are forced to enter cell cycle, cells were co-cultured with CD40L-expressing stroma for 72 h with or without IL-21, and treated with MLN4924 for 8 or 24 h. IL-21 enhanced CLL cell sensitivity to MLN4924. A total of 63.7%±2.6 and 44.4±5.4% underwent apoptosis after a 24 h treatment with 1 *μ*M MLN4924 in the presence or not of IL-21, correspondingly (*P*=0.00004; Figure 1b). Similar findings were observed when CpG-ODN was employed (Supplementary Figure S1A). In contrast, CD3^+^ and CD19^+^ cells isolated from healthy donors exhibited minimal sensitivity to MLN4924 under the same conditions (Supplementary Figure S2A). Thus, CLL cells treated with either IL-21 or CpG-ODN demonstrated enhanced sensitivity to MLN4924.

### MLN4924 deregulates Cdt1 and induces DNA damage and checkpoint activation in CD40L-stimulated CLL cells

**Figure 2:**
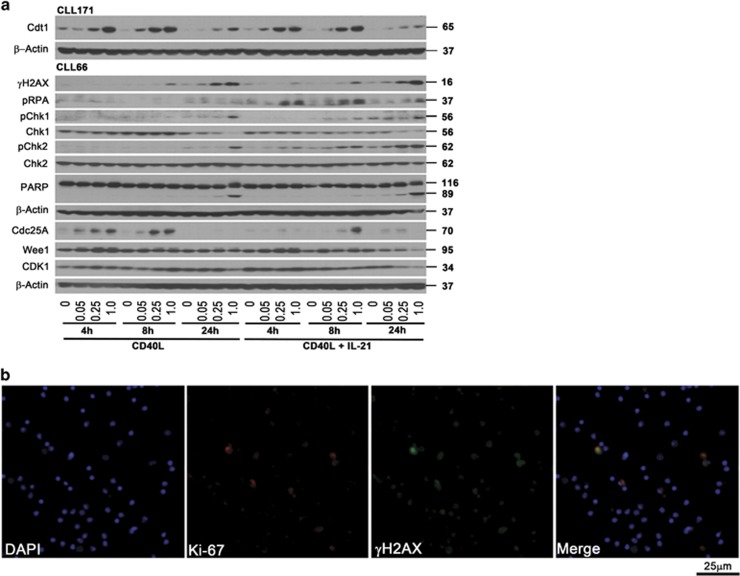
CLL cells undergo DNA damage and checkpoint activation in response to treatment with MLN4924. (**a**) CLL cells were co-cultured with CD40L-expressing stroma in the presence or absence of 25 ng/ml IL-21 for 72 h. Thereafter, cells were treated with 0.05–1 *μ*M MLN4924 or vehicle control, collected at the indicated time points and subjected to immunoblotting. Images representative of four independent experiments are shown. (**b**) CLL cells (*N*=4) were co-cultured as above with 25 ng/ml IL-21 followed by a 6-h incubation with 1 *μ*M MLN4924. Cells were immunostained with *γ*H_2_AX and Ki-67 antibodies. Nuclei were counterstained with 4,6-diamino-2-phenylindole. A representative case is shown

We supposed that the proliferative CLL cell fraction was sensitized to MLN4924. As re-replication is a well-established consequence of NAE inhibition and results from deregulation of Cdt1 function, we then studied Cdt1 in CLL. Indeed, early accumulation of Cdt1 occurred in a dose-dependent manner in the CD40L-stimulated cells treated with MLN4924 (Figure 2a). Cdt1 was also overexpressed in cells stimulated with IL-21 or CpG-ODN (Figure 2a and Supplementary Figure S1B). In contrast, Cdt1 levels remained undetectable in CLL cells cultured off stroma (Supplementary Figure S3). Because IL-21 was more reliable at inducing proliferation than CpG-ODN, we used it in our subsequent experiments.

**Figure 3:**
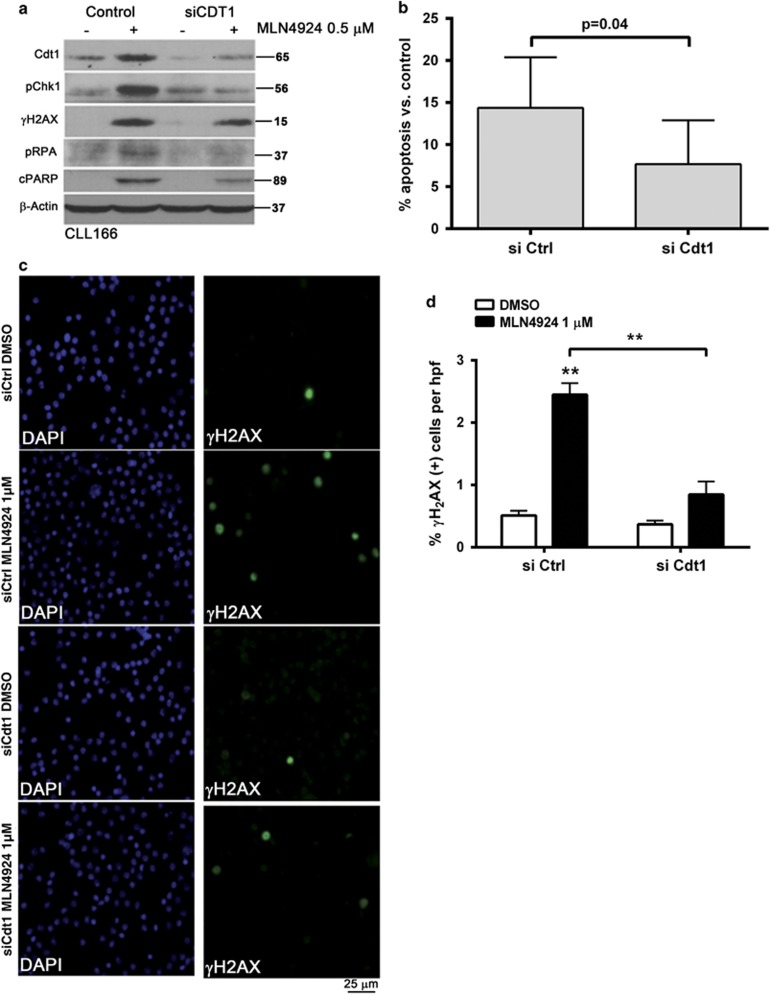
NAE inhibition-induced DNA damage and apoptosis are Cdt1 dependent. CLL cells were transfected with the individual siRNAs against Cdt1 or control siRNA using Amaxa program X-005. Immediately after nucleofection, cells were cultured in the presence of 25 ng/ml IL-21 for 72 h. Thereafter, cells were treated with MLN4924 or vehicle control. (**a** and **b**) Upon 24-h incubation with 0.5 *μ*M MLN4924, proteins were lysed and cells were subjected to immunoblotting. Images representative of four independent experiments are shown. Apoptosis within the CD19^+^ subset of cells (*N*=10) was determined by Annexin V and 7-AAD staining; results were normalized to vehicle-treated control. (**c** and **d**) Upon 6-h incubation with 1 *μ*M MLN4924 (*N*=6), cells were immunostained with *γ*H_2_AX antibodies. A representative case is shown. Six high-power fields per sample were scored for expression of *γ*H_2_AX as described in the Materials and Methods. Data are mean±S.E. **P<0.01 compared with control conditions

As overexpression of Cdt1 has been shown to induce DNA re-replication followed by head-to-tail collision of replication forks,^22^ we tested whether checkpoint activation occurred in CLL cells under these conditions. Indeed, cells treated with MLN4924 exhibited an early rise in markers of DNA damage, such as phosphorylation of H_2_AX (phospho-histone 2A.X) and replication protein A (RPA), and these events preceded caspase cleavage of poly-ADP ribose polymerase (PARP; Figure 2a). These changes were evident at an earlier time point (4 h) in cells co-stimulated with IL-21, likely indicating a higher fraction of cells progressing through cell cycle and thus susceptible to DNA damage. Indeed, we determined that CLL cells undergoing proliferation (as evidenced by expression of Ki-67) succumbed to DNA damage (Figure 2b). In contrast, we observed no evidence of Cdt1 accumulation and DNA damage in normal PBMCs (Supplementary Figure S2B). Furthermore, DNA damage induced checkpoint activation in CLL cells, and this was particularly evident in IL-21-treated cells. Interestingly, both checkpoint kinases 1 and 2 (Chk1 and Chk2) were phosphorylated in the presence of MLN4924, further suggesting that targeting NAE induced activity of both ATM (ataxia telangiectasia mutated) and ATR (ataxia telangiectasia and Rad3 related) activity in CLL cells (Figure 2a). To confirm the role of Cdt1 in checkpoint activation, we performed genetic knockdown of Cdt1 in CD40L/IL-21-stimulated CLL cells and treated them with MLN4924. This manipulation resulted in reduced DNA damage and Chk1 activation as evidenced by immunoblotting and immunostaining, as well as decreased apoptosis (Figure 3 and Supplementary Figure S4).

Degradation of a dual-specificity phosphatase cell division cycle 25A (Cdc25A) involved in G2/S transition is also CRL dependent.^23^ Chk1 phosphorylates and inactivates Cdc25A, thereby inhibiting CDK1 activity and cell cycle progression. Treatment with MLN4924 led to increased Cdc25A levels, whereas the levels of Wee1, another CRL target and a negative CDK1 regulator, did not consistently change in CLL cells in the presence of MLN4924 (Figure 2a).

**Figure 4:**
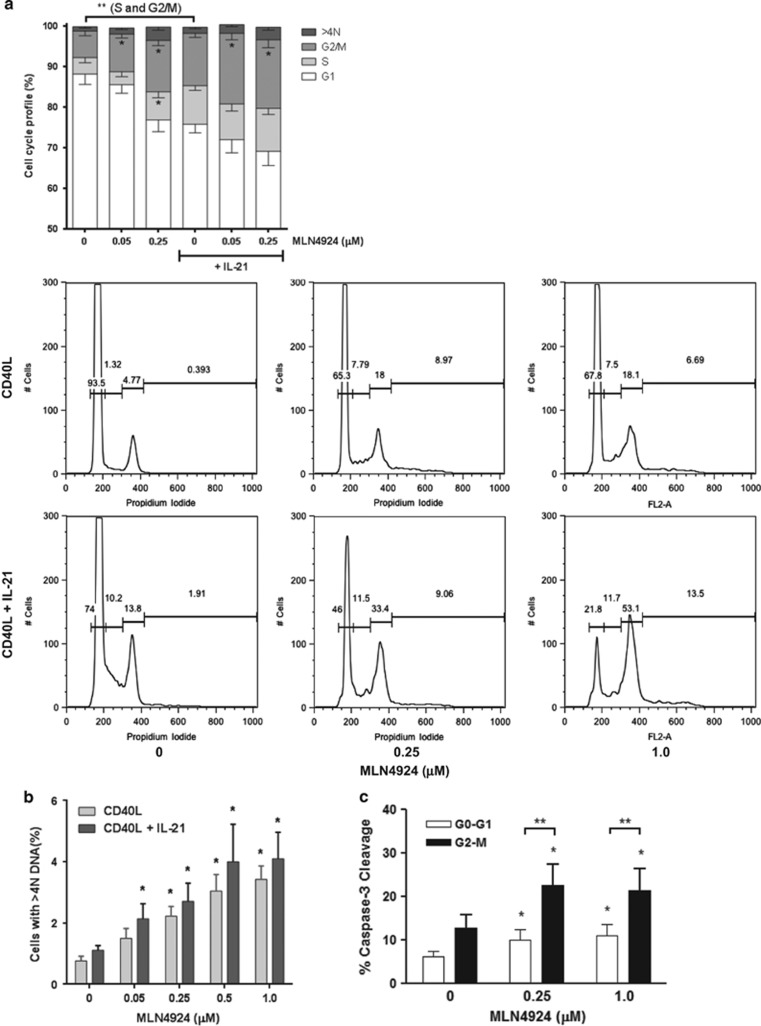
NAE inhibition induces cell cycle deregulation in CLL. (**a** and **b**) CLL cells were co-cultured with CD40L-expressing stroma in the presence or absence of 25 ng/ml IL-21 for 72 h. Cells were treated with the indicated doses of MLN4924 or vehicle control for 24 h and assayed for cell cycle profiling as described in the Materials and Methods. A representative image is shown. (**c**) CLL cells (*N*=6) were cultured as above and assayed for apoptosis (caspase-3 cleavage) within the resting (G_0_/G_1_) and cell proliferative fractions (G_2_/M) after an 8 h exposure to MLN4924. Data are mean±S.E. *P<0.05 and **P<0.01 compared with untreated control

We further analyzed whether Cdt1 accumulation and checkpoint activation in response to NAE inhibition was accompanied by cell cycle deregulation in CD40L-stimulated CLL cells. As expected, stimulation with IL-21 led to an increase in the number of cells in S and G_2_/M phases of cell cycle (Figure 4a; *P*=0.0001 and *P*=0.0003, correspondingly). Furthermore, treatment with MLN4924 caused a pronounced accumulation of cells in G_2_/M phases of cell cycle; an S phase increase was observed in CD40L-only-stimulated cells (Figure 4a). Although CLL cells demonstrated a dose-dependent increase in >4N DNA content, such extensive overreplication was less pronounced in primary lymphoid cells than previously reported in cell lines treated with MLN4924 (Figure 4b).^15^

The above data strongly suggested that Cdt1-mediated DNA re-replication and checkpoint activation contribute to the mechanism of action of MLN4924 in proliferating CLL cells. Therefore, we interrogated CLL cells resident in different phases of cell cycle for early evidence of apoptosis. Indeed, 21.4±5.1% of cells in G_2_/M phases of cell cycle demonstrated caspase-3 cleavage after an 8-h exposure to 1 *μ*M MLN4924 as compared with 10.9±2.6% of cells resting in G_0_/G_1_ (Figure 4c). Thus, targeting NAE in primary CLL cells induces aberrant Cdt1 accumulation with ensuing DNA damage, cell cycle deregulation and accelerated apoptosis of cells induced to proliferate.

### NAE inhibition deregulates endogenous CDK inhibitors in CLL cells

**Figure 5:**
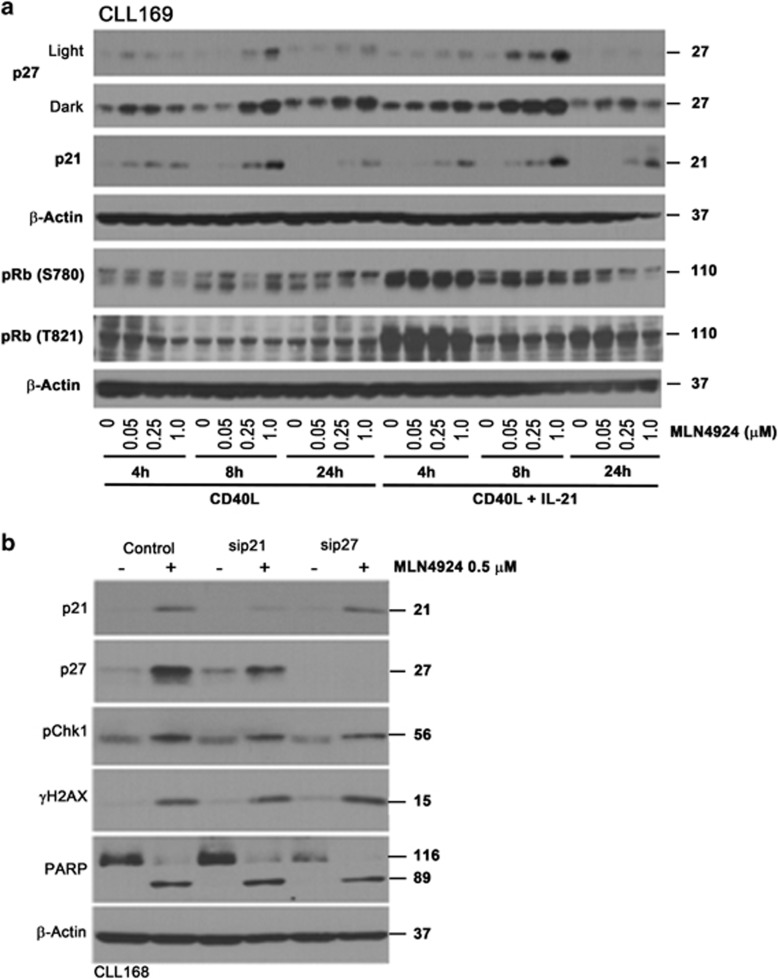
Effect of MLN4924 on CDK inhibitors p21 and p27 in CLL cells. (**a**) CLL cells were co-cultured with CD40L-expressing stroma in the presence or not of 25 ng/ml IL-21 for 72 h. Thereafter, cells were treated with 0.05–1 *μ*M MLN4924 or vehicle control, collected at the indicated time points and subjected to immunoblotting. Images representative of at least four independent experiments are shown. (**b**) CLL cells were transfected with the individual siRNAs against p21, p27 or control siRNA using Amaxa program X-05. Immediately after nucleofection, cells were cultured on CD40L-expressing stroma in the presence of 25 ng/ml IL-21 for 72 h, followed by incubation with 0.5 *μ*M MLN4924 or vehicle control for 24 h. Whole-cell lysates were subjected to immunoblotting. Representative blots of three independent experiments are shown

A number of other relevant proteins regulate cell cycle and are targets of CRL-mediated ubiquitination. p27^Kip1^ and p21^Cip1^ are endogenous CDK inhibitors that bind to Cyclin-CDK2/CDK1/CDK4/6 complexes and thus regulate cell cycle progression at G_1_/S checkpoint. Importantly, p21^Cip1^ also has a defined role in G_2_ checkpoint sustenance after DNA damage.^24^ Hence, we evaluated whether NAE inhibition deregulates endogenous CDK inhibitors, thus enhancing apoptosis in CLL. We found that baseline p27^Kip1^ and p21^Cip1^ protein levels were low in CD40L-stimulated cells, and additional stimulation with IL-21 had no effect (Figure 5a). Following NAE inhibition, both endogenous CDK inhibitors were upregulated in CD40L-stimulated CLL cells, but not in cells cultured off stroma (Figure 5a and Supplementary Figure S3).

As CIP/KIP CDK inhibitors promote dephosphorylated state of pocket proteins (retinoblastoma (Rb), p107 and p130) thereby exercising control over G_1_/S transition, we evaluated whether they might play a role in G_1_/S checkpoint activation in CLL cells treated with MLN4924. Rb protein was hyperphosphorylated in cells stimulated with IL-21, possibly indicating the fact that a larger fraction of cells progressed through cell cycle under these conditions (Figure 5a). We did not observe a change in Rb phosphorylation in CD40L-stimulated cells at early time points of incubation with the drug (4 and 8 h). Meanwhile, late hypophosphorylation of Rb at 24 h could be a result of cell apoptosis (Figure 5a). Finally, knockdown of either p27^Kip1^ or p21^Cip1^ had no effect on *γ*H2AX, Chk1 phosphorylation or cell apoptosis (Figure 5b). Thus, we conclude that the endogenous CDK inhibitors play little role in MLN4924-mediated induction of DNA damage and apoptosis in CLL.

### MLN4924 sensitizes CLL cells to the alkylating agents

We further explored the interaction between MLN4924 and the chemotherapy agents commonly used in treatment of CLL, bendamustine and chlorambucil. Although these alkylating agents induce DNA crosslinking and DNA damage at all phases of the cell cycle, they are most toxic to cells that progress through S phase. As targeting NAE in CLL induces DNA damage, we hypothesized that MLN4924 might sensitize CLL cells to the alkylating agents through this mechanism, where DNA damage might overwhelm DNA repair mechanisms.

**Figure 6:**
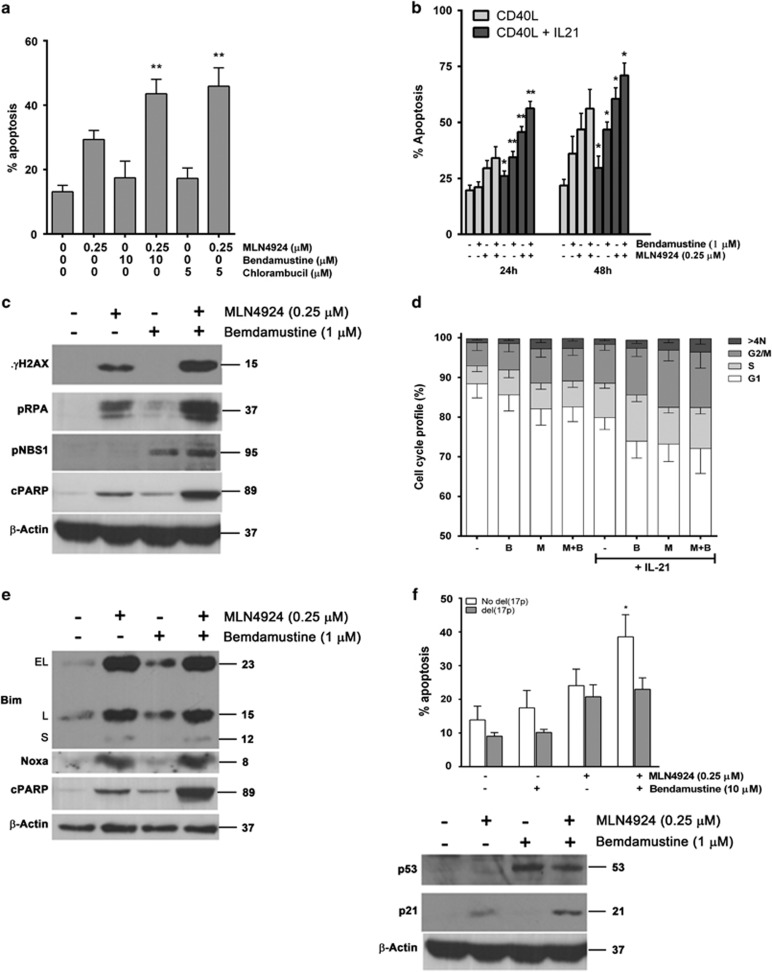
MLN4924 enhances activity of the alkylating agents in CLL. (**a**) CLL cells (*N*=10) were co-cultured with CD40L-expressing stroma for 24 h, followed by incubation with the indicated compounds or with vehicle control for 48 h. Apoptosis within the CD19^+^ subset of cells was determined by Annexin V and 7-AAD staining. (**b**) CLL cells (*N*=8) were co-cultured with CD40L-expressing stroma in the presence or not of 25 ng/ml IL-21 for 72 h. Thereafter, cells were treated as shown. Cells were collected at the indicated time points and assayed for apoptosis (data are mean±S.E.). (**c**) CLL cells were co-cultured with CD40L-expressing stroma for 24 h, followed by incubation with the indicated drugs or with vehicle control for 24 h. Cells were lysed and subjected to immunoblotting (a representative image out of four independent experiments is shown). (**d**) CLL cells (*N*=8) were co-cultured with CD40L-expressing stroma in the presence or not of 25 ng/ml IL-21 for 72 h. Thereafter, cells were treated with drugs alone or in combination for 24 h and assayed by FACS analysis for cell cycle profiling and cell population with more than 4N DNA content. (**e**) CLL cells were co-cultured with CD40L-expressing stroma 24 h, treated with the indicated for 24 h and subjected to immunoblotting. A representative image of three independent experiments is shown. (**f**) Cells with *del(17p)* (*N*=10) or not were treated with drugs as above for 48 h and assayed for apoptosis as above; cells without *del(17p)* were lysed after 24 h of incubation with drugs and subjected to immunoblotting. Data are mean±S.E. **P<0.01 and *P<0.05 compared with either single drug

Although CD40L-stimulated CLL cells were resistant to either bendamustine or chlorambucil alone, treatment with MLN4924 resensitized them to both agents (Figure 6a). Furthermore, the cooperative effect of bendamustine and MLN4924 was enhanced in the presence of IL-21 (Figure 6b). Although treatment with bendamustine alone led to minimal DNA damage in the CLL cells cultured on the CD40L-expressing stroma, both single- and double-strand DNA damage was enhanced in the presence of both drugs, evidenced by phosphorylation of RPA, H2AX and p-NBS1 (Figure 6c). Although re-replication and G_2_ arrest described above were evident in the presence of MLN4924 and, to a lesser extent, bendamustine, the drug combination-mediated DNA damage was not due to an increase in either of these events (Figure 6d).

As we have previously demonstrated that MLN4924 induces BH3-only proteins BIM and NOXA in CLL cells,^4^ and bendamustine induces NOXA in non-Hodgkin's lymphoma cells,^25^ we investigated whether the cooperative effect of these two drugs was due to enhanced upregulation of these pro-apoptotic B-cell lymphoma 2 (BCL2) family members. However, whereas incubation with MLN4924 led to a dramatic induction of NOXA and BIM in CLL cells, bendamustine had no effect, suggesting that this mechanism did not contribute to toxicity of this drug combination (Figure 6e).

Finally, we have previously shown that inhibiting NAE effectively induced apoptosis of CLL cells harboring high-risk features, including *del(17p)*.^4^ Such patients represent an ongoing unmet clinical need.^26^ To determine whether a combination of bendamustine and MLN4924 may represent a promising therapeutic approach in this disease category, we tested cells obtained from patients with *del(17p)* CLL. Although bendamustine has also shown preclinical promise in high-risk CLL,^27^ we did not observe a cooperative effect between the two drugs (Figure 6f). This is consistent with lack of clinical efficacy of bendamustine in CLL with *del(17p)*,^28^ and likely indicates that its cytotoxicity is dependent on functional p53.

## Discussion

A preclinical study by Milhollen *et al.*^8^ provided initial rationale to target neddylation in B-cell malignancies. In line with the context-specific role of neddylation, the cytotoxic effects of MLN4924 in diffuse large B-cell lymphoma (DLBCL) cells were dependent on the cell of origin. In germinal center B-cell-like (GC) DLBCL cells, targeting NAE resulted in accumulation of Cdt1, DNA re-replication and cell cycle arrest in S phase, reminiscent of the consequences of NAE inhibition in adherent human colorectal carcinoma HCT116 cells.^15, 16^ In contrast, in activated B-cell-like (ABC) DLBCL cells, abrogation of transcriptional activity of NF-*κ*B was the dominant event that preceded apoptosis.^8^

We have recently shown that targeting NAE in CLL cells neutralizes NF-*κ*B through disrupted ubiquitination of I*κ*B (canonical pathway) and diminished processing of p100 to p52 (noncanonical pathway), as in ABC DLBCL.^4^ Treatment with MLN4924 shifted the balance of BCL2 family members toward the pro-apoptotic BH3-only proteins, with dramatic upregulation of BIM and NOXA,^4^ an event of high importance in CLL cells whose survival is highly dependent on the anti-apoptotic BCL2 family members.^29^ Disruption of NF-*κ*B activity as a consequence of NAE inhibition is therefore an important mechanism of MLN4924-induced apoptosis in activated CLL cells that received stimulation with CD40L or BAFF (B-cell activating factor) in the stromal niche.^30, 31^ However, niche-resident CLL cells are exposed to a variety of stimuli beyond those necessary for NF-*κ*B activation and demonstrate decreased apoptotic priming, that is, higher threshold of sensitivity to apoptosis via intrinsic mitochondrial pathway,^18^ and hence upregulation of the pro-apoptotic BH3-only proteins may be less deadly.

Although proliferation of the CLL cells in peripheral circulation is negligible,^32^ clone renewal may be substantial,^33^ suggesting that cells found in the CLL proliferation centers may be susceptible to MLN4924-mediated cell cycle deregulation. Here we extend our earlier findings to ascertain that Cdt1 accumulated in CD40L-activated CLL cells treated with MLN4924. Ensuing re-replication^22^ leads to DNA damage and checkpoint activation, contributing to MLN4924 toxicity in CLL. As S-phase cells demonstrate enhanced susceptibility to MLN4924-induced DNA re-replication,^15^ we stimulated CLL cells with IL-21,^21^ significantly expanding proliferative cell fraction, and thus were able to sensitize CLL cells to MLN4924. A larger proportion of cells showed evidence of DNA damage and cell cycle arrest when coincubated with IL-21, potentially relevant to cells induced to proliferate by their microenvironment *in vivo*. Importantly, our data also implicate that changes in culture conditions can switch the cell fate from an NF-*κ*B inhibition program to a Cdt1 induction program when NAE is inhibited, as both phenomena are observed on the same cell background (primary malignant B cell).

We observed that CLL cells predominantly arrested in G_2_ upon treatment with MLN4924. In contrast, some DLBCL cells underwent S-phase arrest.^8^ Interestingly, a recent study suggested that lower concentrations of MLN4924 induce G_2_ arrest, whereas saturating doses of the drug cause a delay in S-phase progression.^23^ Genetic knockdowns of Cdt2, a conserved component of CRL4^Cdt2^ E3 ligase that targets Cdt1 for degradation, or of geminin, a negative regulator of Cdt1, lead to G_2_ arrest.^34, 35^ Thus, different means of inducing re-replication may result in activation of either intra-S or G2 checkpoints. It is also possible that the S-phase arrest observed in DLBCL cells could also have resulted from disrupted ubiquitination of the CDK inhibitors p21^Cip1^ and p27^Kip1^. In the CLL cells, these proteins accumulated but did not alter cell fate in response to MLN4924, although their role in G2/S transition has not been fully explored in this study.

Earlier findings suggested that CLL cells possess DNA damage repair ability that is highly variable between individual samples.^36, 37^ However, understanding of DNA repair mechanisms in CLL is limited as it was only studied in resting cells *ex vivo*. In our work we demonstrate that CLL cells, when forced to enter cell cycle in stromal co-cultures, are able to activate DNA damage response. Generation of single- and double-stranded DNA breaks, as evident by RPA phosphorylation, *γ*H2-AX in response to treatment with MLN4924 and phospho-NBS1 (predominantly in response to bendamustine) resulted in activation of Chk1 and Chk2 kinases. Although bendamustine was minimally toxic in stromal co-cultures, checkpoint activation was not sufficient to repair DNA damage induced by the NAE inhibitor. High expression of endogenous BCL2 may inhibit ribonucleotide reductase activity, thereby reducing the pool of intracellular dNTPs.^38^ CLL cells are known to express high levels of BCL2 protein, and hence may be particularly sensitive to DNA damage. We therefore argue that NAE inhibition-mediated replication stress may contribute to increased apoptotic priming of the CLL cells.

Despite significant advances of targeted therapy in CLL, chemoimmunotherapy remains a mainstay of treatment in younger patients, particularly in the upfront setting. Bendamustine, a bifunctional alkylating agent, has received widespread use in CLL.^28, 39, 40^ Bendamustine induces double-strand DNA damage, purportedly its main mechanism of toxicity, as well as unique DNA repair responses such as base excision repair.^25, 41^ In lymphoma cell lines, bendamustine activated p53 via elevated expression and phosphorylation at S15,^25^ a target site of ATM, ATR and DNA-PK. In accordance with these data, we observed phosphorylation of NBS1 (Nijmegen breakage syndrome 1) in response to bendamustine treatment in CLL cells, consistent with double-stranded DNA damage. Despite that, CLL cells were resistant to bendamustine on stroma in the absence of IL-21, indicating that sufficient DNA repair took place. However, a combination of MLN4924 and bendamustine significantly augmented the extent of double-stranded DNA damage and led to enhanced apoptosis. As bendamustine is particularly efficient in cycling cells, IL-21 co-stimulation sensitized CLL cells to the drug combination. Finally, NAE inhibition was shown to impair the DNA damage response,^17, 42^ providing further potential explanation for the cooperative action of this drug combination in CLL. Our findings echo a recent preclinical analysis where bendamustine-induced DNA damage was enhanced because of fludarabine-mediated inhibition of DNA repair and abrogated the protective effects of stroma.^43^ Importantly, we show that MLN4924 in combination with bendamustine did not enhance apoptosis of the CLL cells with *del(17p)*, suggesting that although this is a promising therapeutic strategy, it is not relevant to CLL with defective p53.

In summary, we demonstrate that targeting NAE induces DNA damage, checkpoint activation and cell cycle arrest in CLL cells. This, coupled with abrogation of the NF-*κ*B activity, results in apoptosis and reversal of stromal-mediated protection. Our study sheds light on the mechanism of action of MLN4924 in CLL and B-cell malignancies and suggests a new therapeutic strategy to enhance the efficacy of the alkylating agents.

## Materials and methods

### Patient samples, CLL and stromal cell co-cultures

Peripheral blood was obtained from patients with B-CLL at Dartmouth-Hitchcock Medical Center and Oregon Health and Science University following approval by the respective institutional review board and written informed consent of patients, the majority of whom were previously untreated. Blood was also obtained from six healthy volunteers. Isolation of peripheral blood mononuclear cells (PBMCs) was performed using standard Ficoll-Hypaque techniques (Amersham, Piscataway, NJ, USA). Such CLL samples had >90% CD5^+^/CD19^+^ cells, as determined by flow cytometry. CLL cells were cultured in RMPI-1640 supplemented with 15% fetal bovine serum, 100 U/ml penicillin, 100 *μ*g/ml streptomycin, 2 mM L-glutamine, 25 mM HEPES, 100 *μ*M non-essential amino acids and 1 mM sodium pyruvate (Lonza, Walkersville, MD, USA). Ten CLL samples with 17p deletion were obtained from the CLL Center at Dana-Farber Cancer Institute. All experiments were performed with freshly isolated CLL cells except the viability assays involving the latter that were performed with viably frozen cells.

The mouse fibroblast cell line (L cells) engineered to express CD40L (L4.5) was given to us by Dr. Sonia Neron (Université Laval, Laval, QC, Canada)^44^ and were maintained in RPMI-1640 medium with 10% FBS and penicillin–streptomycin. CLL cells were cultured under standardized conditions on stromal cells as previously described.^4^ Briefly, stromal cells were seeded to achieve 80–100% confluence; on the following day, CLL cells were plated at a 50 : 1 ratio and incubated at 37 °C in 5% CO_2_. To induce proliferation, CLL cells were stimulated with 25 ng/ml IL-21 (Cell Signaling, Danvers, MA, USA) or 1.5 *μ*M CpG-ODN (a kind gift from Dr. John Byrd, Ohio State University, Columbus, OH, USA) for 72 h, treated with drugs thereafter and assayed for apoptosis, cell cycle distribution or immunoblotting as detailed below. At harvest, CLL cells were gently washed off the stromal layer. When collected for protein analysis, CLL cells were transferred to a new plate and incubated for an additional 60 min to allow reattachment of stromal cells and thus minimize contamination of CLL cells. Where indicated, cells were also cultured off stroma at the same density.

### Cell viability testing and drugs

CLL cell apoptosis was measured in duplicate as previously described using the ApoScreen Annexin V Apoptosis Kit.^45^ Briefly, cells were resuspended in 150 *μ*l of Annexin V binding buffer containing 1 *μ*l of Annexin V-PE, 1 *μ*l of 7-aminoactinomycin (7-AAD) and 1 *μ*l of CD19-FITC (Southern Biotech, Birmingham, AL, USA) and CD3-APC antibodies (Miltenyi Biotec, San Diego, CA, USA), followed by flow cytometry on a MACSQuant (Miltenyi Biotec). The following drugs were used: MLN4924 (provided by Millennium Pharmaceuticals Inc., Cambridge, MA, USA), bendamustine, BMS-345541 (Sigma-Aldrich, St. Louis, MO, USA) and chlorambucil (MP Biomedicals, Solon, OH, USA).

### Immunoblotting

Cells were lysed in RIPA buffer (20 mM Tris, 150 mM NaCl, 1% NP-40, 1 mM NaF, 1 mM sodium phosphate, 1 mM NaVO3, 1 mM EDTA, 1 mM EGTA, supplemented with protease inhibitor cocktail (Roche, Indianapolis, IN, USA), phosphatase inhibitor cocktail 2 (Sigma-Aldrich) and 1 mM phenylmethanesulfonyl fluoride). Proteins were analyzed by immunoblotting as previously described.^45^ The following antibodies were used: Bim, CDC25A, Chk1, phospho-Chk1 (S345), *γ*H2AX (S139), PARP and cleaved PARP, p21, p53, phospho-p95/NBS1 (S343), Rb (S780), Wee1 (all from Cell Signaling); Cdt1 (F-6), p27 (C-19), total Rb (C-15; Santa Cruz Biotechnology, Santa Cruz, CA, USA), NOXA (Imgenex, San Diego, CA, USA), Rb (T821; Life Technologies, Carlsbad, CA, USA), *β*-actin (Sigma-Aldrich), phospho-RPA32 (S4/S8) (Bethyl, Montgomery, TX, USA) and horseradish peroxidase-conjugated anti-mouse and anti-rabbit antibodies (Bio-Rad, Hercules, CA, USA).

### Immunocytochemistry

3 × 10^5^ cells were adhered onto polylysine D-coated coverslips (Sigma-Aldrich) during a 45-min incubation at 37 °C, fixed in 10% formalin (Fisher Scientific, Pittsburgh, PA, USA) and permeabilized in 1% Triton-X 100 in PBS. Coverslips were blocked for 30 min in 5% bovine serum albumin (Sigma-Aldrich) in PBS with 0.1% Tween-20, probed with *γ*H_2_AX (S139), phospho-RPA32 (S4/S8) or Ki-67 antibodies (conjugated with AlexaFluor647; Life Technologies) and then with DyLight 488 goat anti-rabbit antibodies (Thermo Scientific, Carlsbad, CA, USA). Coverslips were mounted with anti-fading ProLong Gold Solution (Life Technologies) with 4′,6-diamidino-2-phenylindole (DAPI, for nuclear counterstaining). Fluorescent images were captured with a Zeiss AxioCam MRm camera mounted on a Zeiss Observer.Z1 microscope (Zeiss, Jena, Germany). Cells positive for *γ*H_2_AX or phospho-RPA were counted in six high-power fields per condition ( × 20 magnification) and referenced to the total number of cells quantified using ImageJ software (National Institutes of Health, Bethesda, MD, USA).

### siRNA-mediated gene silencing

Electroporation of siRNA into CLL cells was performed using Amaxa Human B-cell Nucleofection Kit (Amaxa, Cologne, Germany) as previously described.^45^ Briefly, 1 × 10^7^ PBMCs were mixed with 100 *μ*l of Amaxa B-cell nucleofector solution, and 2 *μ*g of siRNA was nucleofected using program X-05. This resulted in transfection efficiency of 30–60% (as determined with 2 *μ*g pMaxGFP plasmid) and viability of 50–80% cells at 24 h. The following siRNA sequences were used (sense strand): Cdt1, 5′-GCAAUGUUGGCCAGAUCAA-3′ p27, 5′-GCAACCGACGAUUCUUCUACUCA-3′ p21, 5′-CUGUACUGUUCUGUGUCUU-3' (all from Dharmacon, Lafayette, CO, USA).

### Cell cycle analysis

2 × 10^5^ cells were fixed in ice-cold 70% ethanol while being vortexed, incubated on ice for 15 min, washed in PBS and resuspended in 250 *μ*l of staining solution containing 20 ng/ml propidium iodide, 200 ng/ml RNAse A (Sigma-Aldrich), 0.1% Triton-X 100 and 1 *μ*l CD19-FITC mAb in PBS. Cells were incubated for 15 min and submitted to flow cytometry. For concurrent cell cycle and apoptosis staining, 5 × 10^6^ cells were fixed and permeabilized using the Inside Stain Kit (Miltenyi Biotec) according to the manufacturer's instructions. Staining solution contained 20 ng/ml propidium iodide, 200 ng/ml RNAse A, 1 *μ*l anti-cleaved caspase-3 antibody (Cell Signaling) and 1 *μ*l DyLight-488 goat anti-mouse secondary antibody (Thermo Scientific). Cell cycle analysis was performed using FlowJo software (Tree Star, Ashland, OR, USA).

### 5-ethynyl-2′-deoxyuridine (EdU) incorporation analysis

At 2 h before collection, cells were treated with EdU, a thymine analog, to allow for incorporation in replicating cells. Cells were then collected, washed with PBS, fixed in 2% paraformaldehyde and stored at 4 °C until analysis. Subsequently, cells were processed using Click-iT EdU AlexaFluor 647 Assay Kit (Life Technologies) according to the manufacturer's instruction, and subjected to flow cytometry. Analysis was performed using the FlowJo software.

### Statistical analysis

Results of individual experiments were analyzed using paired and unpaired Student's *t*-test, Fisher's exact test, nonparametric Mann–Whitney test and Spearman's *r*. Statistical analyses were completed using the GraphPad Prism 6 software package (La Jolla, CA, USA). All tests were two sided, and data were considered to be statistically significant at *P*<0.05.

## Abbreviations

CD40L: CD40 ligand
Cdt1: chromatin licensing and DNA replication factor 1
IL-21: interleukin-21
CpG-ODN: CpG oligodeoxynucleotide
*γ*H2AX: phospho-histone 2A.X
Chk: checkpoint kinase
Cdc25A: cell division cycle 25A
CDK: cyclin-dependent kinase
NBS1: Nijmegen breakage syndrome 1
Bcl2: B-cell lymphoma 2
PARP: poly-ADP ribose polymerase
Rb: retinoblastoma
