# Supplementary figures and images for: Targeting neddylation induces DNA damage and checkpoint activation and sensitizes chronic lymphocytic leukemia B cells to alkylating agents
Source: Cell Death Dis. 2015 Jul 9;6(7):e1807–. doi: 10.1038/cddis.2015.161 (PMC4650717; doi:10.1038/cddis.2015.161)

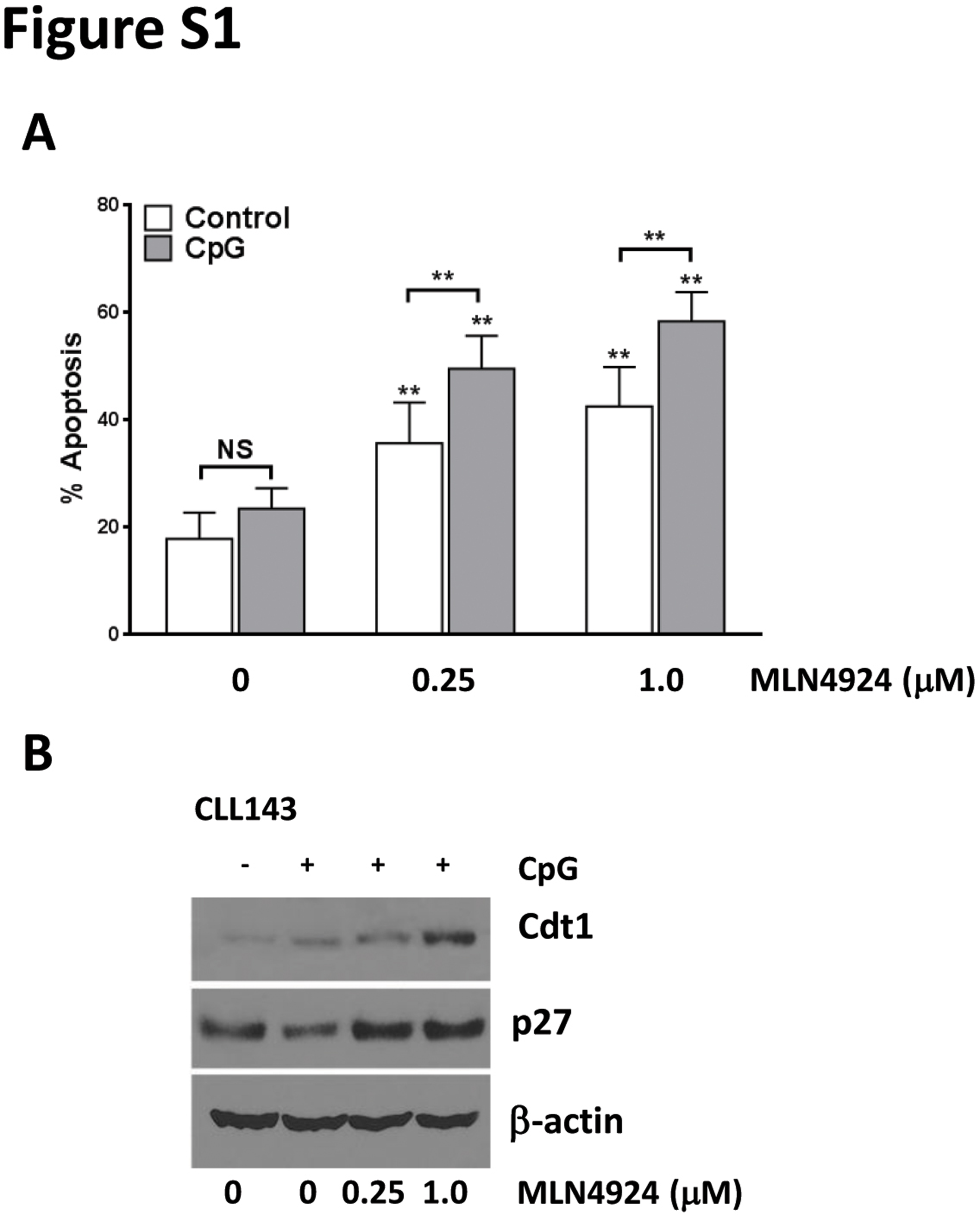

Supplement: Supplementary Figure 1 [file cddis2015161x1.tif]

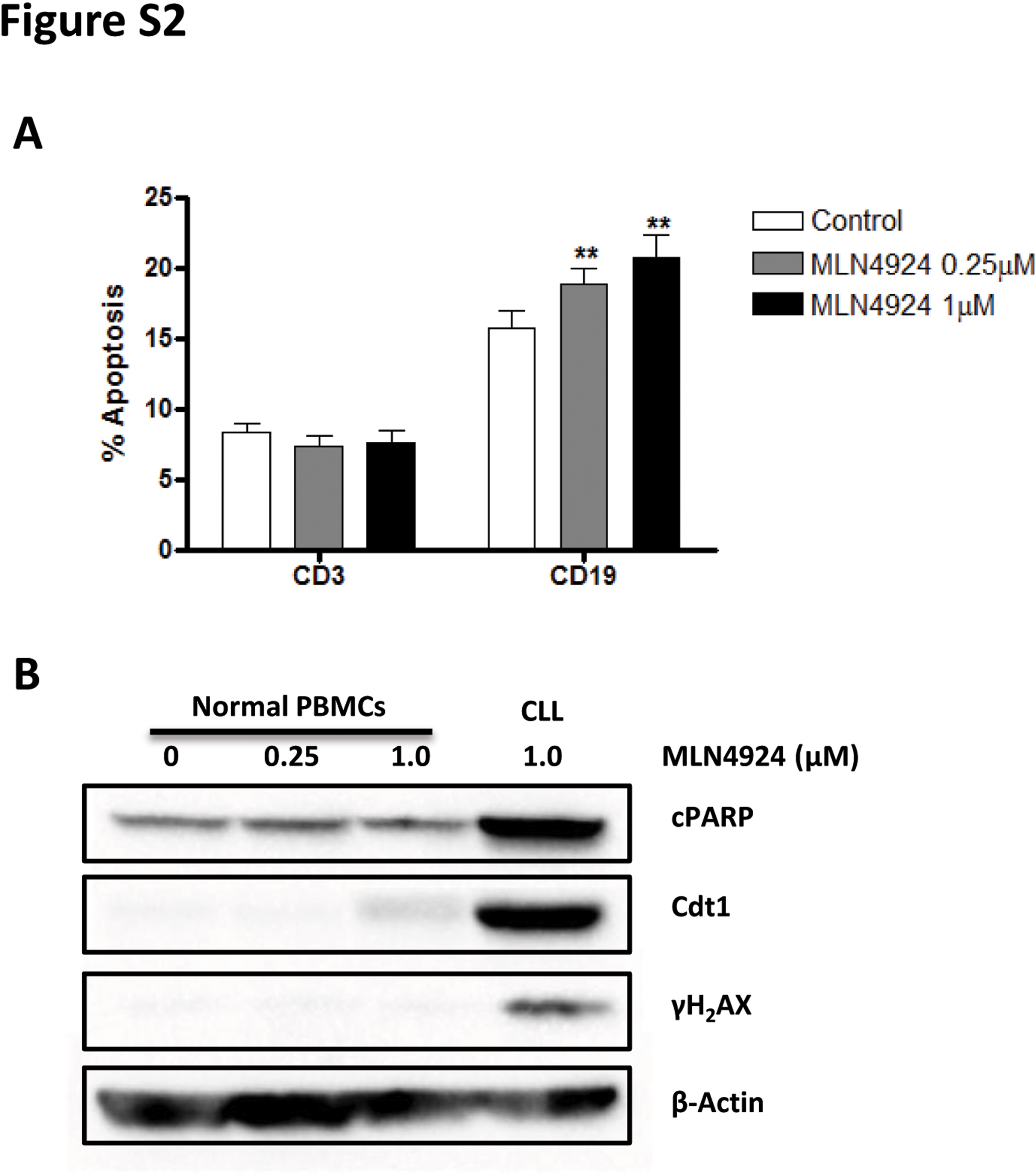

Supplement: Supplementary Figure 2 [file cddis2015161x2.tif]

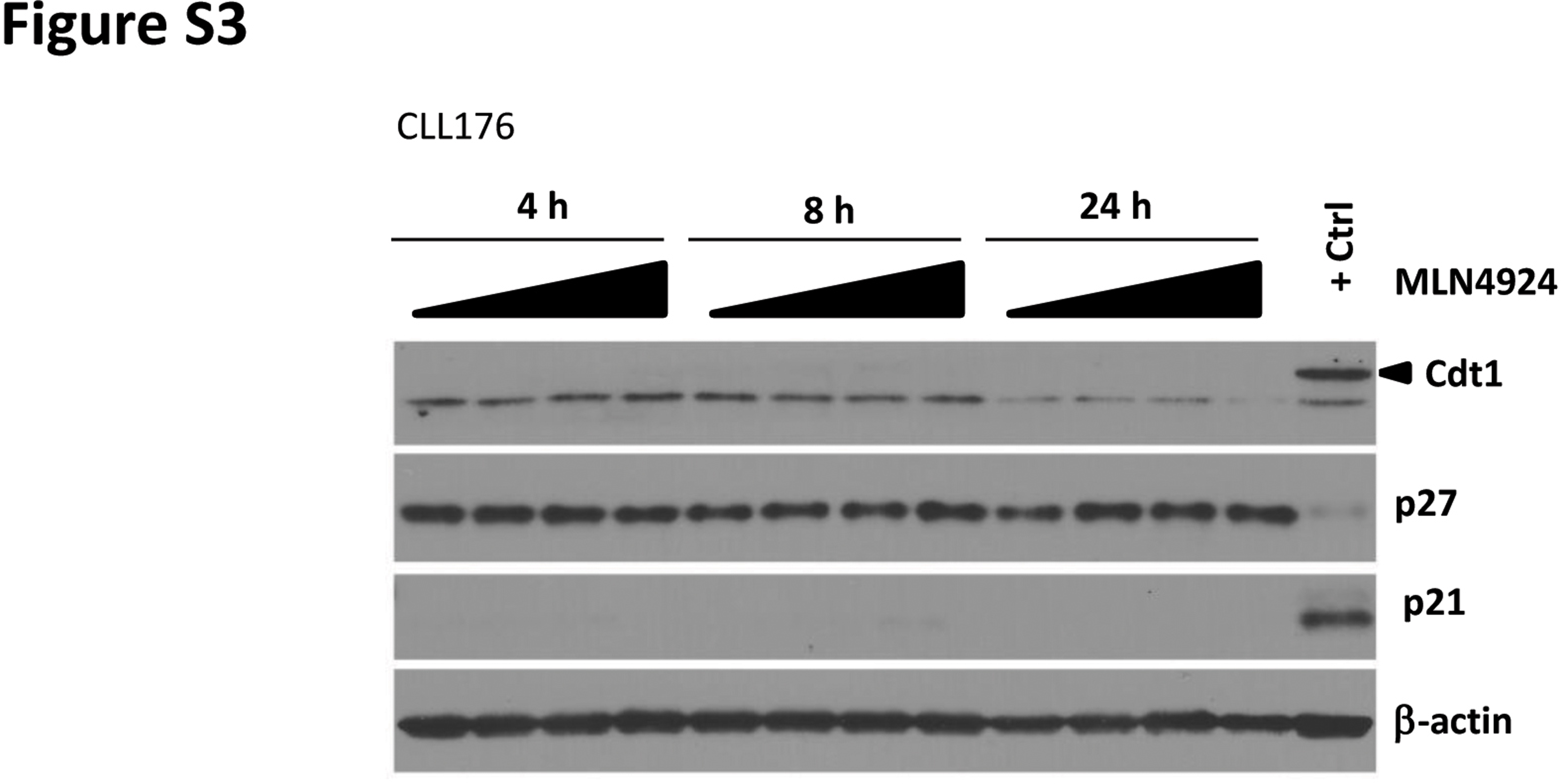

Supplement: Supplementary Figure 3 [file cddis2015161x3.tif]

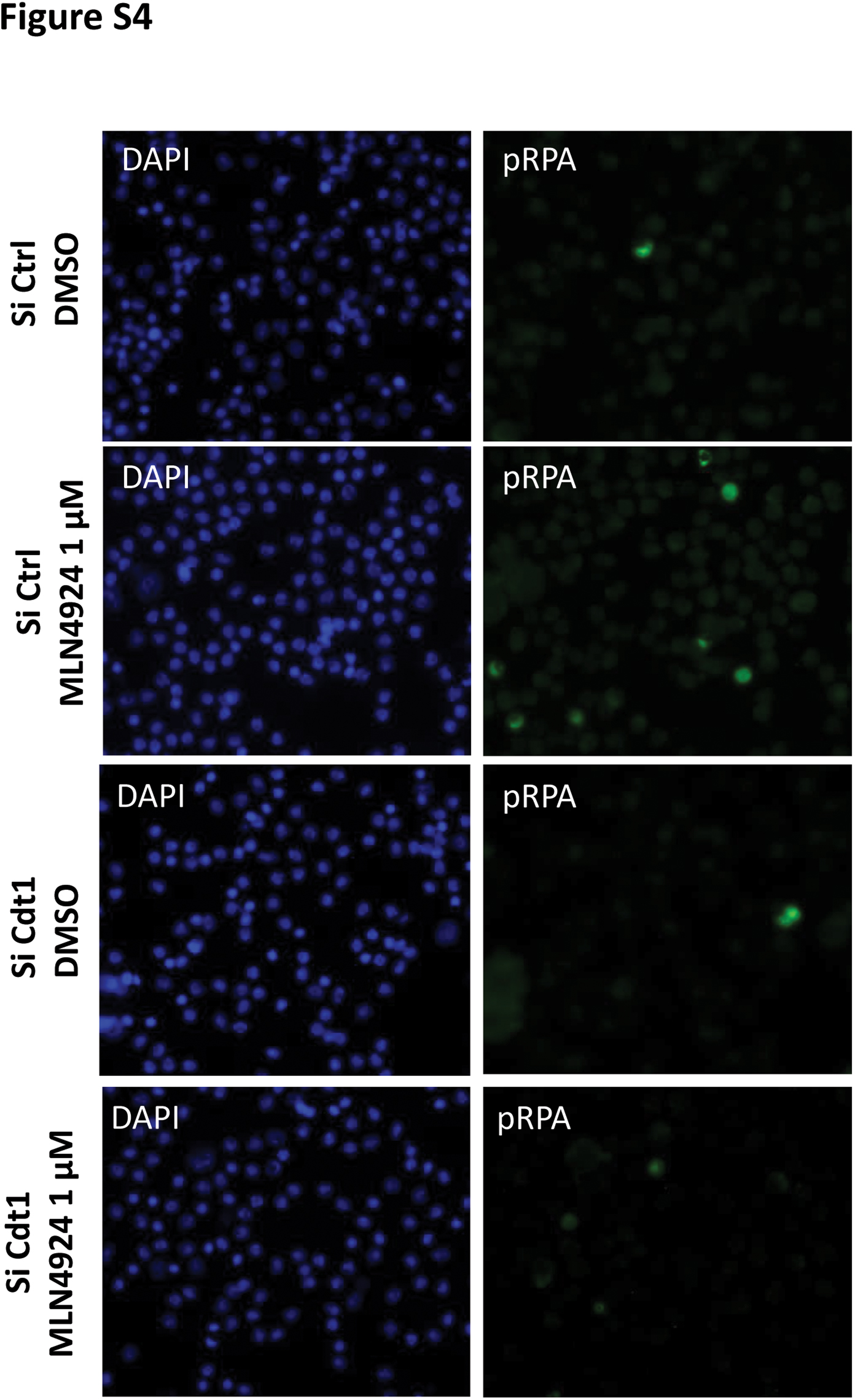

Supplement: Supplementary Figure 4 [file cddis2015161x4.tif]
