# Supplementary material for: Targeting neddylation induces DNA damage and checkpoint activation and sensitizes chronic lymphocytic leukemia B cells to alkylating agents
Source: Cell Death Dis. 2015 Jul 9;6(7):e1807–. doi: 10.1038/cddis.2015.161 (PMC4650717; doi:10.1038/cddis.2015.161)
Supplement: Supplementary Figure Legends [file cddis2015161x5.pdf]

Supplementary Figure legends.

Supplementary Figure S1. Treatment with CpG-ODN sensitizes CLL cells to NAE inhibition. CLL cells were co-cultured with CD40L-expressing stroma in the presence or not of 1.5 µM CpG-ODN. After 48 hours, cells were treated with the indicated doses of MLN4924 or vehicle control for 24 hours. *(A)* Apoptosis within the $CD19^+$ subset of cells (N=6) was determined by Annexin V and 7-AAD staining. Data are mean±SEM. ** - p<0.01 compared to control conditions. *(B)* Cells were lysed and subjected to immunoblotting. A representative image from 1 of 4 experiments is shown.

Supplementary Figure S2. NAE inhibition in PBMCs. PBMC's isolated from healthy donors were co-cultured with CD40L-expressing stroma and 25 ng/mL Il-21 for 72 hours. Cells were treated with 0.25 - 1 µM MLN4924 or vehicle control for 24 h. *(A)* Apoptosis within the $CD19^+$ and $CD3^+$ subset of cells (N=6) was determined by Annexin V and 7-AAD staining. Data are mean±SE. ** - p<0.01 compared to control conditions. *(B)* Cells were lysed and subjected to immunoblotting. A representative image from 1 of 4 experiments is shown. Right lane is a CLL sample treated similarly.

Supplementary Figure S3. Targeting NAE does not induce Cdt1, p21 or p27 accumulation in peripheral blood CLL cells cultured off stroma. CLL cells were incubated with 0.05, 0.25 or 0.5 µM MLN4924 or vehicle control for 2 - 24 hours. Whole-cell protein lysates were subjected to immunoblotting. A representative image from 1 of 3 experiments is shown. As a reference, cells were also treated with 0.25 µM MLN4924 for 8 hours on CD40L-expressing stroma (right lane - +Ctrl).

Supplementary Figure S4. Genetic knockdown of Cdt1 counters MLN4924-mediated DNA damage in CLL cells. CLL cells (N=6) were transfected with the individual siRNA's against Cdt1 or control siRNA using Amaxa program X-05. Immediately after nucleofection, cells were cultured in the presence of 25 ng/mL IL-21 for 72 hours. Thereafter, cells were treated with 1 µM MLN4924 or vehicle control for 6 hours and immunostained with phospho-RPA antibodies. Nuclei were counterstained with 4,6-diamino-2-phenylindole. A representative case is shown.
